# The benefits and risks of maternal RSV vaccination on mortality in South Africa: A modeling study

**DOI:** 10.1371/journal.pmed.1004625

**Published:** 2026-01-20

**Authors:** Ayaka Monoi, Akira Endo, Simon R. Procter, Sequoia I. Leuba, Stefan Flasche, Mark Jit, Maternal RSV Vaccine Benefit-Risk Advisory Group

**Affiliations:** 1 Department of Infectious Disease Epidemiology and Dynamics, London School of Hygiene & Tropical Medicine, London, United Kingdom; 2 Centre for Mathematical Modelling of Infectious Diseases, London School of Hygiene & Tropical Medicine, London, United Kingdom; 3 School of Tropical Medicine and Global Health, Nagasaki University, Nagasaki, Japan; 4 Charité Center for Global Health, Charité—Universitätsmedizin Berlin, Berlin, Germany; 5 Saw Swee Hock School of Public Health, National University of Singapore, Singapore, Singapore; 6 Department of Global and Environmental Health, School of Global Public Health, New York University, New York, New York, United States of America; Makerere University College of Health Sciences, UGANDA

## Abstract

**Background:**

Maternal respiratory syncytial virus (RSV) vaccine, RSV prefusion F protein vaccine (RSVpreF (Abrysvo)), was found to be safe and efficacious in the MATISSE trial. However, post-hoc stratified analyses identified an excess of preterm births in the intervention arm in two upper-middle-income countries, most prominently in South Africa. This study weighs the potential benefits and risks in mortality associated with maternal RSV vaccination in South Africa, assuming the increased risk of preterm births observed in the trial was caused by vaccination.

**Methods and findings:**

We compared the estimated RSV-associated infant deaths averted by vaccination (benefits) and neonatal mortality potentially associated with vaccine-associated risk in preterm birth (risks) in South Africa. The benefit model estimated the South African RSV disease burden in 2011−2016 and waning vaccine protection during infancy. The risk model estimated excess neonatal mortality using gestational age (GA)-specific mortality data from the Drakenstein Child Health Study and the GA-specific birth distribution in South Africa in the MATISSE trial, but did not incorporate the mortality risk found in the MATISSE vaccine trial in which no excess deaths occurred.

The benefit model estimated that vaccination would reduce RSV-associated infant deaths by 31 (95% credible interval (Crl): 27, 35) per 100,000 live births born to vaccinated mothers in South Africa. Using the number of infants born to mothers vaccinated at 24–36 GA weeks in the MATISSE trial, if the association in South Africa between vaccination and preterm birth is actually causal, the risk model suggested that neonatal deaths would increase by 44 (95%CrI: −43, 210), totaling 1.4 (95%CrI: −1.4, 6.9) excess neonatal deaths for every infant RSV death prevented. When this was changed to the number of infants born to mothers vaccinated at 27–36 GA weeks in the MATISSE trial, the predicted risks dropped and in 97% of the simulations the benefits outweighed the risks. The outcome was sensitive to the GA window that we used to determine which infants to include in the analysis. The study was limited by only considering mortality associated with RSV disease and preterm birth.

**Conclusions:**

If RSVpreF increases preterm birth risk, and if this increases neonatal mortality, then the benefit-risk analysis failed to show that the direct benefits of vaccination in reducing RSV-associated infant mortality would substantially outweigh the risks of preterm birth-associated neonatal mortality in South Africa with vaccination from 24 GA to 36 GA weeks. There was large uncertainty in the analyses due to small numbers of preterm births. With vaccination from 27 GA weeks, the benefit-risk analysis favored vaccination. RSVpreF vaccination has the potential to be safe and effective when used from the third trimester.

## Author summary

**Why was this study done?:**

- RSVpreF, a maternal RSV vaccine, was licensed in some countries after its phase III trial showed efficacy.
- An unplanned analysis showed a statistically significant increase in preterm delivery risk among vaccinees in South Africa.
- An informed recommendation needed to be made by the World Health Organization Strategic Advisory Group of Experts on Immunization regarding the use of maternal RSV vaccine in countries where mortality risk of infant RSV disease and neonatal mortality are relatively high.

**What did the researchers do and find?:**

- We developed a static cohort model to estimate potential benefits and risks of maternal RSV vaccination in South Africa.
- For the benefit estimate, we estimated RSV-associated deaths averted by vaccination.
- For the risk estimate, we estimated excess neonatal deaths potentially associated with vaccination, assuming the link is causal.
- We estimated 1.4 (95%CrI: −1.4, 6.9) excess neonatal deaths for every infant RSV death prevented, using trial birth data of infants born to mothers given vaccine or placebo from 24 weeks of gestational age.
- We found that using trial birth data of infants born to mothers given vaccine or placebo from 27 weeks of gestational age, the estimated risk decreased so that in 97% of our simulations the benefit exceeds the risk.

**What do these findings mean?:**

- Maternal RSV vaccination from the third trimester has the potential to be safe and effective.
- Including other outcomes than mortality in benefit-risk analysis may influence decision-making.

## Abstract 3

Ayaka Monoi and colleagues model the benefits and risks of maternal RSV vaccination by gestational age and show that benefits outweigh risks with vaccination from the third trimester.

## Introduction

Respiratory syncytial virus (RSV) is a significant cause of pediatric morbidity and mortality worldwide, particularly in low- and middle-income countries (LMICs) [1]. It is estimated that globally, RSV leads to approximately 100,000 deaths among children under 5 years in a year [1]. The burden is concentrated in LMICs where over 97% of RSV-attributable deaths occur [1], and especially high among younger infants, who are at the greatest risk of developing severe disease [1–4]. Antillón and colleagues estimated that although children under 6 months of age represent only 10% of the under 5 years population in LMICs, they bear a disproportionate burden of disease, accounting for approximately 30% of hospitalizations and 38%−50% of deaths [3].

Prophylactics against RSV in infancy have recently been licensed, including a maternal RSV vaccine [5] and a long-acting monoclonal antibody [6]. The bivalent RSV prefusion F protein-based vaccine (RSVpreF) developed by Pfizer (Abrysvo, hereafter referred to as “RSVpreF”) has received approval for use in pregnant women from the United States Food and Drug Administrationand the European Medicines Agency (EMA) following the successful completion of the MATISSE trial [5] (hereafter referred to as “the trial”). The trial provided evidence of the vaccine’s efficacy in preventing RSV disease in infants, especially severe disease. Mathematical modeling studies suggested that an RSV vaccination programme could reduce RSV-associated mortality and be cost-effective, particularly in LMICs like South Africa [7–9]. However, in the trial, a non-significant imbalance in preterm birth rates was observed: overall this was not statistically significant with 5.7% (95% confidence interval (CI): 4.9, 6.5) of infants being born prematurely in the intervention arm versus 4.7% (95%CI: 4.1, 5.5) in the placebo arm [10]. The imbalance in preterm birth rates was most pronounced in South Africa [10,11]. Post-hoc analysis found that there is a statistically significant increase of preterm birth risk in the intervention arm in the South African component of the trial (relative risk between the intervention arm and the placebo arm is 2.06 (95%CI; 1.21, 3.51)) [12]. However, no imbalance in newborn and infant deaths between study arms was observed either in the whole trial or in South Africa (e.g., for the whole trial, 8 in the intervention arm versus 14 in the placebo arm with relative risk of 0.57 (95%CI: 0.24, 1.36)) [10,12,13]. Nevertheless, the observed imbalance in preterm birth rates has led to concerns about vaccine safety. Notably, a trial of RSVpreF3-MAT vaccine in pregnant women was terminated due to increased preterm birth rates in the intervention arm, accompanied by a non-significant numerical increase in neonatal deaths likely related to prematurity [14]. To inform national decision-making, it is crucial to balance the risks of maternal RSV vaccination against the benefits [15] in the local context.

This study aimed to assess the potential impact of maternal RSV vaccination with RSVpreF in South Africa on mortality, should the excess risk of preterm birth associated with vaccination be substantiated and should preterm births translate into mortality. Given the low relative risk of preterm births recorded among the trial participants in Argentina, Chile, the United States, Taiwan, and Japan [13], similar analyses for those countries would result in the risks being very unlikely to exceed the benefits after the actual preventive effect of vaccination on RSV is included. We compare the vaccine benefits of reducing RSV-associated infant mortality against the potential risks of increased neonatal mortality due to preterm birth in the most pessimistic scenario.

## Methods

### Ethical approvals

Ethical approval was obtained from the Faculty of Health Sciences Human Research Ethics Committee, University of Cape Town (401/2009) and the Western Cape Provincial Health Research committee for using the Drakenstein Child Health Study (DCHS) data for additional studies such as this one. In addition, the use of these data for this study received ethical approval from the London School of Hygiene & Tropical Medicine Ethics Committee (Ref: 29955).

### Model outline

To assess the risks of RSVpreF associated with the potential safety signal from the South African component of the trial, for the purpose of this analysis, we assumed that maternal RSV vaccination is causally associated with an increased risk of preterm birth, which contributes to an increased risk of neonatal mortality. It is important to note that it is currently impossible to assess the true nature of this potential safety signal with any certainty and that we are not trying to do this here, instead we investigate the hypothetical mortality implications if the signal were to be confirmed subsequently.

**Fig 1:**
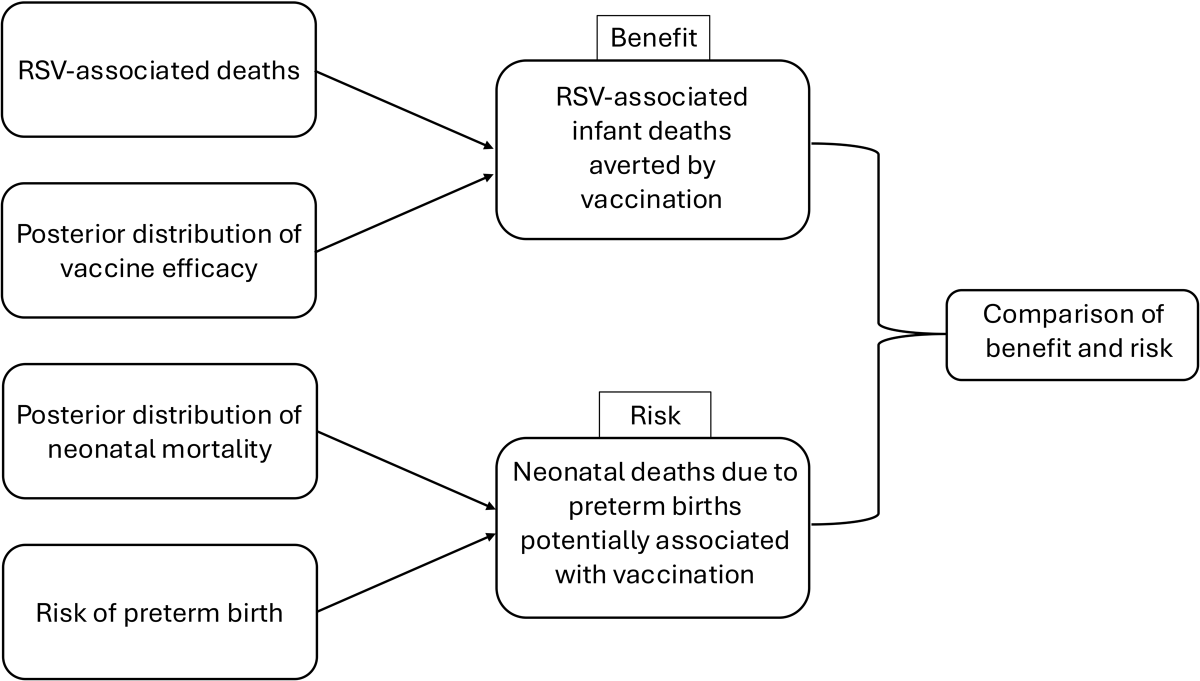
Overview of the modeling framework. Our study follows this workflow.

We quantified the benefits measured as the number of RSV-associated deaths prevented in the first year of life, and the risk as the number of potential excess neonatal deaths associated with preterm birth per 100,000 live births born to South African mothers vaccinated with RSVpreF (Fig 1). The phase III trial birth data used in this study can be downloaded from [13]. The phase III trial efficacy data were obtained from [11]. Data from the DCHS [16] were obtained from Heather Zar. Analysis was performed using R version 4.3.3. [17]

### Benefits

Vaccine-preventable RSV-associated infant deaths were estimated using a previously described model of vaccine impact [8] with updated assumptions on vaccine efficacy and its waning. In brief, age-specific South African RSV-associated deaths were estimated in the model, using the baseline RSV incidence from South Africa in 2011–2016. Deaths averted through vaccination during the first year of life were then estimated by multiplying the number of RSV-associated deaths by age-specific vaccine efficacy. Given that antibody titers among preterm infants born to vaccinated mothers were still several-fold higher than those among preterm and term infants born to mothers given placebo, we assumed uniform vaccine protection among vaccinees, regardless of their gestational ages (GAs) [18–20]. We also assumed that there were no indirect effects of vaccination, i.e., that maternal vaccines do not affect the overall transmission dynamics of RSV beyond the vaccinated mother and her infant.

#### RSV-associated infant mortality.

RSV-associated infant mortality in South Africa has been estimated in our static cohort model [8]. We first estimated age-stratified RSV-associated severe acute respiratory illness (SARI) rates in South Africa using country-specific surveillance and ecological data in 2011–2016 [4]. We then estimated RSV-associated infant deaths using the SARI hospitalization rates, in-hospital case fatality rate (CFR), and accounted for underreporting due to out-of-hospital deaths estimated from national vital statistics data [21].

To assess the uncertainty in our estimates, we performed a probabilistic sensitivity analysis. Assuming normally-distributed errors around the reported estimates of RSV-associated SARI hospitalizations, we fitted the distributions to the median and 95% credible intervals (Crls) from our previous model [4,8]. We generated 10,000 samples per age group and used these in estimating the median and 95%Crls of the benefit endpoints.

#### Efficacy and waning of vaccine protection.

Our previous model [8] assumed that vaccine efficacy was either constant or waned exponentially over its duration of protection, based on fitting to trial data available at that time. Given the observed vaccine efficacy waning in the trial [5,11], we re-estimated vaccine efficacy and waning of protection from the trial data using the following Bayesian framework. We assumed that vaccine efficacy against severe and less severe RSV-associated medically-attended (MA) lower respiratory tract infections (LRTIs) [11] wanes following an Erlang-2 distribution. We sampled parameters to reflect a consistent rate of waning protection across both outcomes, while allowing the initial strength of protection to potentially differ for each outcome. To characterize waning vaccine-derived immunity after birth, we fitted the model jointly to the trial observations for severe and less severe RSV-associated MA-LRTIs [11] using Markov chain Monte Carlo (MCMC). We used the observations from 0 to 180 days after birth grouping into 30-day intervals as presented by Munjai and colleagues [11]. The Crls for the number of averted deaths include both the uncertainty from estimated RSV CFR and that of the fitted vaccine efficacy. Further details are described in Section D in S1 Text.

### Risks

Fatal outcomes among preterm births were rare in the trial: in the South African component of the trial, two neonatal deaths in the intervention arm and two in the placebo arm were reported during follow-up [13]. To estimate excess neonatal deaths potentially associated with vaccination, we instead used GA-specific neonatal mortality estimates from a South African longitudinal birth cohort study [16] and combined those with the probability of delivery at a specific GA in the trial. We constructed distributions of GA at delivery for vaccinated and unvaccinated mothers using the trial data. We then calculated expected excess deaths using differences in GA-specific delivery proportions among vaccinated and unvaccinated mothers, and neonatal mortality estimated from Zar and colleagues [16].

#### Gestational age-specific delivery risk of vaccinees.

The difference in delivery proportions at each GA among vaccinated and unvaccinated mothers was calculated from the trial observations [13]. The primary analysis was conducted using all GA at birth data as observed in the South African component of the trial. We conducted scenario analyses by excluding early preterm births (<34 weeks) in the trial. We also estimated the risk when using only trial data for live births born to mothers vaccinated at 27–36 gestational weeks in the South African component. This analysis attempts to simulate what might happen if vaccination was only delivered within this GA window (not just by excluding early preterm infants).

To account for the uncertainty in GA distributions, we bootstrapped (10,000 iterations) the number of births by trial arm in South Africa, focusing on GAs at which infants were born.

#### Gestational age-specific neonatal mortality.

Using a Bayesian framework, we estimated GA-specific neonatal mortality risk in South Africa from data from the DCHS [16], a longitudinal birth cohort study, in which from 2012 to 2015 women were enrolled in their second trimester of pregnancy from peri-urban health facilities. Recorded outcomes include live births as well as neonatal deaths stratified by GA, specifically <28 weeks, 28, 29, 30, 31, 32, 33, 34, 35, 36, and 37+ weeks gestation.

We fitted an Erlang-2 distribution to data on neonatal mortality at 28–36 weeks to obtain a smooth function of GA-specific neonatal mortality. Outside this range, because of lack of disaggregated data, we assumed that for births born before 28 weeks, the neonatal mortality was the same as that at 27 weeks. For infants born after 36 weeks, instead of assuming uniform distribution of the data among the subgroup, we assumed the number of births follows the distribution in the South African component of the phase III trial [13] and all the deaths observed in the South African cohort study were infants born at 37 weeks, i.e., the earliest GA in that subgroup. We also assumed that neonatal mortality risk was constant from 37 weeks onwards.

We fitted the function to the births and deaths using MCMC. We also conducted a scenario analysis using pooled birth and death data from the Vulnerable Newborn Measurement Collaboration, a study combining population-based data from 15 LMICs from 2000 to 2017 [22] instead of the aforementioned assumption for constant neonatal mortality for infants born after 36 weeks gestation as neonatal mortality likely varies by GA (Section A3 in S2 Text).

GA dating in DCHS was performed by second-trimester ultrasonography which is consistent with the trial where most participants GA was established by the second trimester ultrasound [16]. We bootstrapped GAs in Zar and colleagues assuming GA assessment by second-trimester ultrasonography has an accuracy of ±14 days [23]. This was assumed to be uniformly distributed. The details are described in Section A4 in S2 Text.

### Scenario analysis

Extremely and very preterm births (born before 32 weeks gestation) were rare (<0.5% of all births) in both placebo and intervention arms of the trial [13]. We performed scenario analyses to investigate how influential these small numbers of early preterm births were. We estimated excess neonatal mortality in South Africa under different assumptions regarding data from the trials: (i) excluding the earliest birth in each trial arm (i.e., 27 weeks in the intervention arm and 30 weeks in the placebo arm), and (ii) excluding the five earliest births in each trial arm.

We performed a scenario analysis with the highly optimistic assumption that vaccine efficacy remains 80% for the first year of life (Section A2 in S2 Text).

## Results

### Infant deaths averted through vaccination

Estimated rate of underlying RSV-associated hospitalized SARI in South Africa was 39,000 (95%Crl: 36,000, 42,000) per 100,000 person-years and estimated rate of RSV-associated deaths was 51 (95%Crl: 47, 55) per 100,000 person-years.

**Fig 2:**
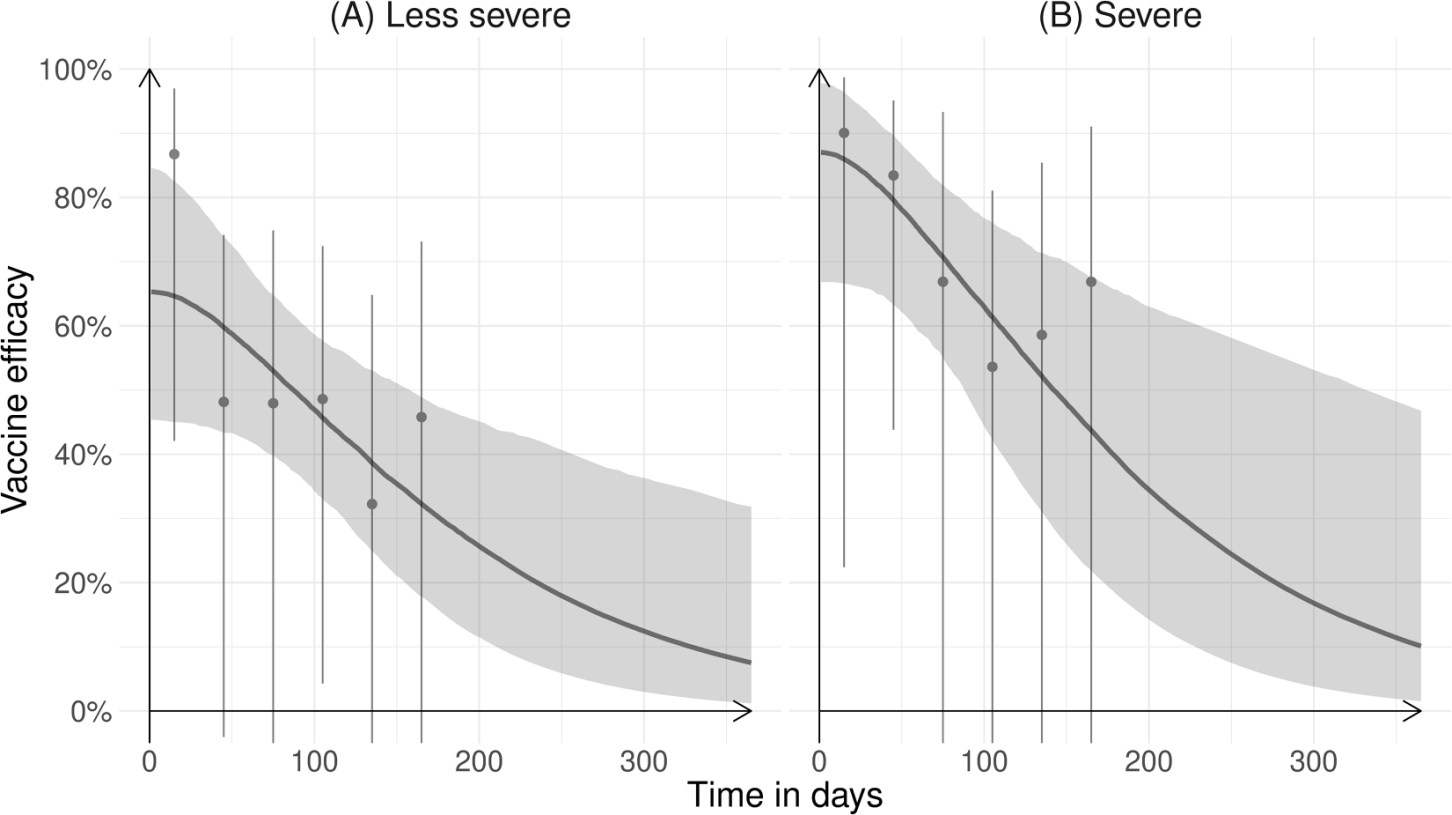
Vaccine efficacy of RSVpreF against RSV-associated (A) less severe and (B) severe MA-LRTIs during the first year of life. Vaccine efficacy against RSV-associated severe MA-LRTIs observed in the trial is shown as gray dots together with binomial 95% confidence intervals. Modeled efficacy is shown as a gray line with gray-shaded 95% credible intervals.

The modeled efficacy against severe RSV-associated MA-LRTIs was 87% (95%Crl: 67, 98) on the first day of life and that against less severe RSV-associated LRTI was 65% (95%Crl: 45, 85). Efficacy waned to 10% (95%Crl: 1.6, 47) and 7.5% (95%Crl: 1.2, 32) a year following birth, respectively (Fig 2).

Using this vaccine efficacy in the impact model, we estimated that maternal RSV vaccination would prevent 31 (95%Crl: 27, 35) RSV-associated infant deaths per 100,000 live births born to vaccinated mothers in South Africa.

### Neonatal deaths potentially associated with vaccination

**Fig 3:**
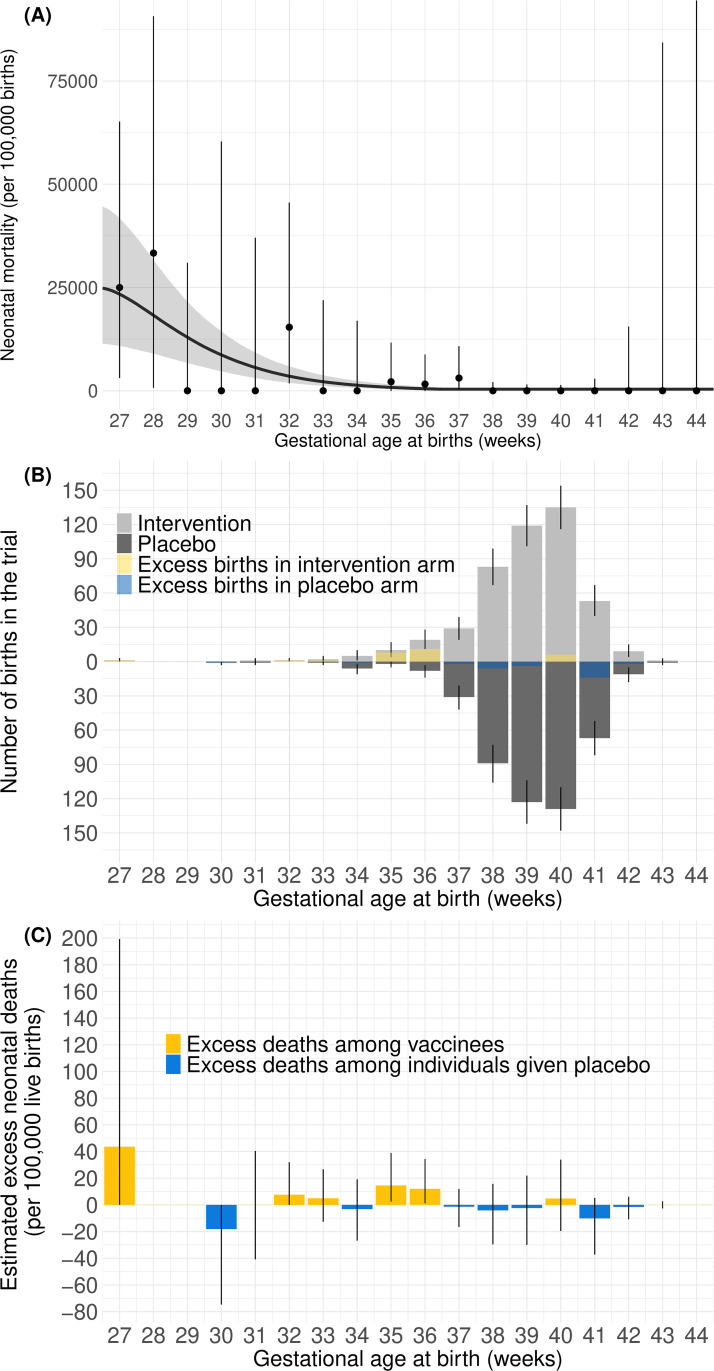
Gestational age (GA)-specific estimates of neonatal mortality in South Africa. **(A)** Neonatal mortality per 100,000 live births by GA at birth from Zar and colleagues [16]. Observed mortality is shown as dots together with binomial 95% confidence intervals. Modeled neonatal mortality is shown as a gray curve line with gray-shaded 95% credible intervals. **(B)** Bootstrapped GA-specific births born to mothers vaccinated or given placebo at 24–36 GA weeks in the South African component of the trial by trial arm. Resampling trial birth observations with replacement, light and dark gray bars indicate the median of bootstrapped numbers of births in the intervention and placebo arms, respectively. The yellow and blue bars show the difference in medians of numbers of births at each GA. Yellow bars indicate a higher median in the intervention arm, while blue bars indicate a higher median in the placebo arm. Error bars show 95% uncertainty range. **(C)** Estimated GA-specific excess neonatal deaths per 100,000 live births born to mothers vaccinated or given placebo at 24–36 GA weeks. Bars show estimated excess neonatal deaths at each GA. If modeled deaths are larger among newborns born to vaccinated mothers, the bars are colored yellow. If modeled deaths are larger among newborns born to unvaccinated mothers, the bars are colored blue. Error bars show 95% credible intervals.

We modeled neonatal mortality risk as a function of GA week using data from the large South African birth cohort study [16], we estimated that in South Africa neonatal mortality within the first 28 days of life if born at 37 weeks of gestation or later was 370 (95%Crl: 170, 800) per 100,000 live births (Fig 3A). Neonatal mortality increased to 3,700 (95%Crl: 2,000, 6,100) per 100,000 live births if born at 32 weeks and 24,000 (95%Crl: 11,000, 42,000) at 27 weeks. Combining the modeled neonatal mortality and the observed GA at birth in the trial arms (Fig 3C), we estimated that the excess neonatal mortality risk associated with preterm birth risk was 44 (95%CrI: −43, 210) per 100,000 live births.

We applied the model-estimated neonatal mortality risks in the primary analysis to the number of live births observed in the two trial arms in the South African component. We estimated that 2.0 and 2.3 neonatal deaths would have occurred among infants born to unvaccinated and vaccinated mothers, respectively. This compares to 2 observed neonatal deaths in both the placebo and intervention trial arms. Similarly, applying the model-estimated RSV-associated risk for infant deaths to the number of live births in each South African trial arm, we estimated 0.24 and 0.096 infant deaths among infants born to unvaccinated and vaccinated mothers, respectively. This compares to no RSV-associated infant deaths observed in either trial arm.

**Fig 4:**
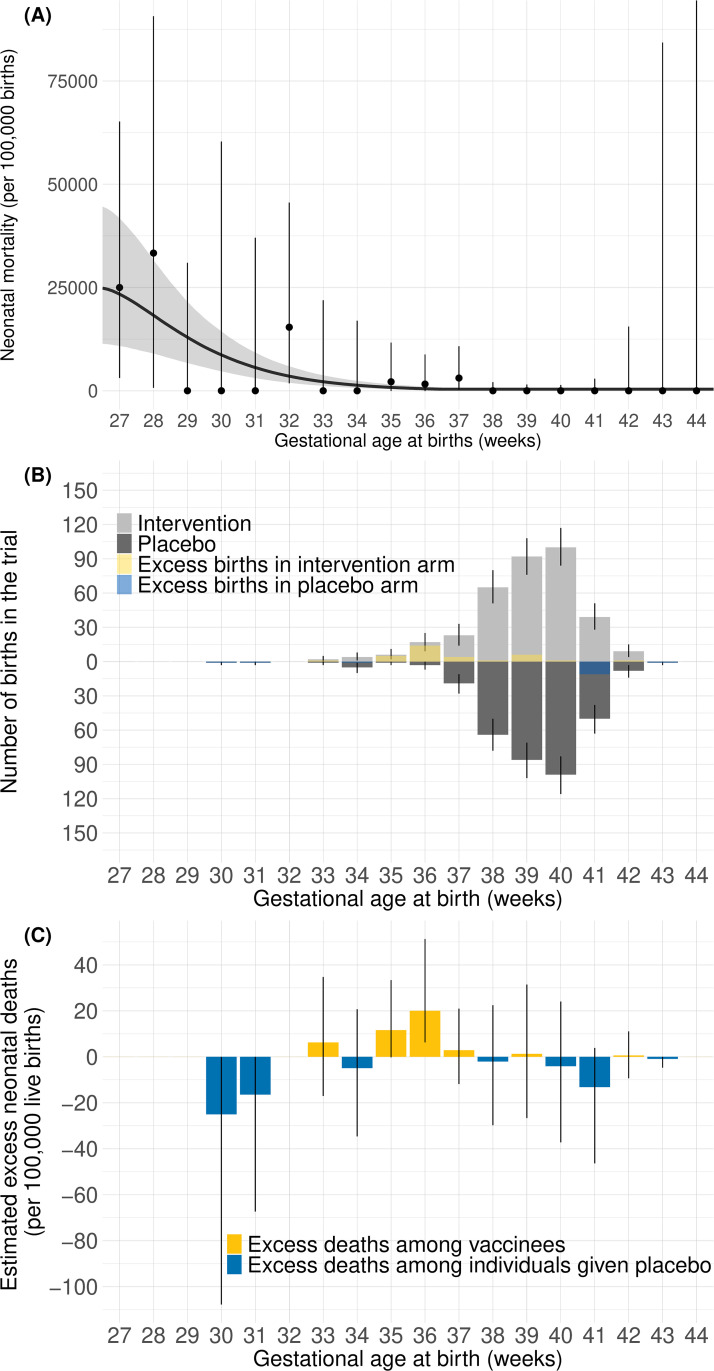
Gestational age (GA)-specific estimates of vaccination-associated neonatal mortality in South Africa using trial outcomes of live births born to mothers vaccinated or given placebo at 27–36 GA weeks in the South African component. **(A)** Neonatal mortality per 100,000 live births by GA at birth from Zar and colleagues [16]. Observed mortality was shown as dots together with 95% confidence intervals. Modeled neonatal mortality is shown as a gray curve line with gray-shaded 95% credible intervals. (**B)** Bootstrapped GA-specific births born to mothers vaccinated or given placebo at 27–36 GA weeks in the South African component of the trial by trial arm. Resampling trial birth observations with replacement, light and dark gray bars indicate the median of bootstrapped numbers of births in the intervention and placebo arms, respectively. The yellow and blue bars show the difference in medians of numbers of births at each GA. Yellow bars indicate a higher median in the intervention arm, while blue bars indicate a higher median in the placebo arm. Error bars show 95% uncertainty range. (**C)** Estimated GA-specific excess neonatal deaths per 100,000 live births born to mothers vaccinated or given placebo at 27–36 GA weeks. Bars show estimated excess neonatal deaths at each GA. If modeled deaths are larger among newborns born to vaccinated mothers, the bars are colored yellow. If modeled deaths are larger among newborns born to unvaccinated mothers, the bars are colored blue. Error bars show 95% credible intervals.

However, when we restricted our analyses to include only trial data for mothers vaccinated at 27 GA weeks onwards, 111 infants in the intervention arm and 131 in the placebo arm were excluded. We then estimated the risk to be −24 (95%Crl: −120, 31) neonatal deaths per 100,000 live births born to vaccinated mothers; i.e., a net reduction in neonatal mortality based on differences in preterm birth among trial arms (Fig 4B).

### Comparison of benefit and risk

For South Africa, we estimated that with vaccination in 24−36 GA weeks, there may be an excess of 13 (95%Crl: −75, 180) neonatal deaths per 100,000 live births associated with maternal RSV vaccination (Table 1). For every infant (between birth and 12 months) saved through protection against RSV by maternal vaccination, there may be 1.4 (95%CrI: −1.4, 6.9) excess neonatal deaths associated with potentially vaccine-associated preterm birth. In 41% of our simulations, the estimated benefit exceeded the estimated risk. In 22% of our simulations, at least five infant deaths were prevented for each neonatal death associated with vaccine-associated preterm birth (Table 1, Fig D in S1 Text).

**Table 1:** Risks and benefits by scenario.

Scenario	Risk (excess neonatal deaths due to vaccine-associated preterm birth per 100,000 live births born to vaccinated mothers) Median (95%Crl)	Benefit (vaccine-preventable RSV-associated infant deaths per 100,000 live births born to vaccinated mothers) Median (95%Crl)	Net mortality (= risk – benefit) per 100,000 live births born to vaccinated mothers) Median (95%CrI)	Risk-benefit ratio (Excess neonatal deaths (risk) per 1 infant saved by vaccination (benefit)) Median (95%Crl)	Percentages of simulations where the benefit exceeds the risk	Percentages of simulations where the benefit exceeds 5 times the risk
Base case	44 (−43, 210)	31 (27, 35)	13 (−75,180)	1.4 (−1.4, 6.9)	41%	22%
27–36 GA weeks vaccination	−24 (−120, 31)	31 (27, 35)	−54 (−150, 0.37)	−0.76 (−4.0, 1.0)	97%	84%
Scenario analysis						
Earliest birth removed	21 (−20, 78)	31 (27, 35)	−9.7 (−52, 46)	0.69 (−0.66, 2.5)	66%	23%
Earliest 5 births removed	15 (−0.42, 39)	31 (27, 35)	−16 (−32, 8.5)	0.48 (−0.014, 1.3)	92%	15%

The risk is measured by excess neonatal deaths due to vaccine-associated preterm birth per 100,000 live births born to vaccinated mothers, and the benefit is measured by the vaccine-preventable RSV-associated infant deaths per 100,000 live births born to vaccinated mothers. “Base case” presents the estimates using all births in the South African component of the trial. “27–36 GA weeks vaccination” presents the estimate using trial outcomes of infants born to mothers vaccinated (or given placebo) at 27–36 weeks. “Earliest birth removed” and “Earliest 5 births removed” present the estimates using births in the South African component without the first earliest birth in each arm (i.e., 27 weeks in the intervention arm and 30 weeks in the placebo arm), and estimates using births in South African component without the 5 earliest births in each arm, respectively. For the four scenarios, the benefits are estimated using all births in South Africa in the trial (i.e., vaccinated at 24–36 weeks).

**Fig 5:**
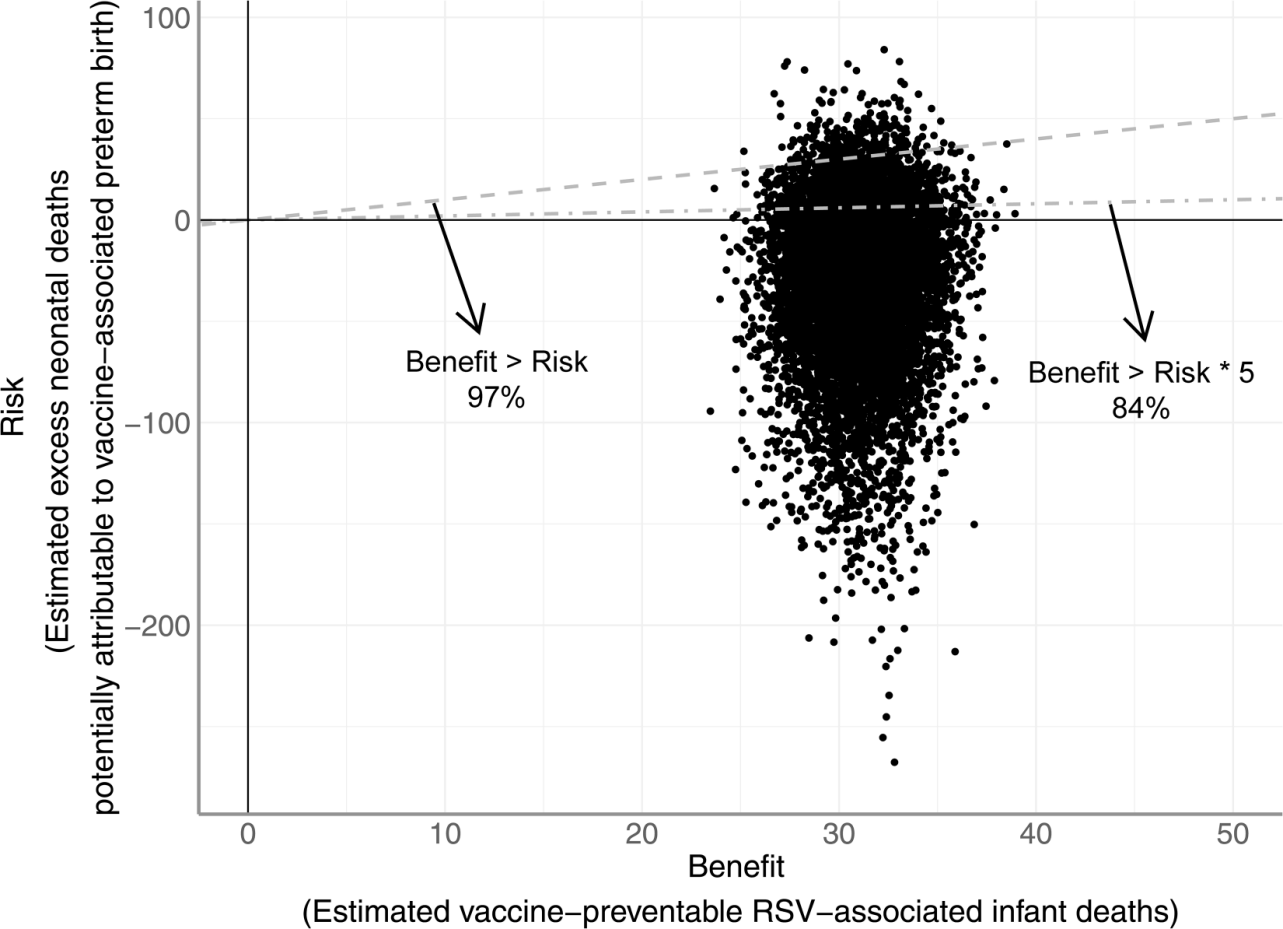
Estimated risks of RSVpreF in South Africa using trial birth outcomes born to mothers vaccinated (or given placebo) at 27–36 GA weeks compared to estimated benefits. The risk is measured by the excess neonatal deaths potentially attributable to vaccine-associated preterm birth estimated using trial birth data of infants born to mothers vaccinated (or given placebo) at 27–36 GA weeks (subset of data). The benefit is measured by the number of vaccine-preventable RSV-associated infant deaths in less than 1-year-old infants using vaccine efficacy estimated from trial data of infants born to mothers vaccinated at 24–36 GA weeks (full dataset). Dots indicate posterior samples of the estimated risk and benefit. Dots below the dashed line indicate that benefit exceeds risk in that simulation (i.e., more than one life would be saved per every one life lost). Dots below the dot-dash line indicate that the benefit exceeds five times the risk in that simulation (i.e., more than five lives would be saved per every one life lost). Percentages besides the lines indicate the percentage of simulations that exceeds the benefit-risk ratio of 1:1 and 5:1, respectively.

With risk estimated using data for infants born to mothers vaccinated or given placebo at 27−36 GA weeks, the estimated benefit exceeds the risk by 54 (95%Crl: −0.37, 150). In 97% of simulations, the estimated benefit exceeds the estimated risk, and in 84% of simulations, the benefit exceeded the risk by more than a factor of five (Table 1, Fig 5).

### Scenario analysis

#### Exclusion of early births.

In our modeled results, a small number of very early preterm births in the trial data substantially influenced the estimated benefit-risk ratio (Fig 3B and 3C). When the earliest birth in each arm (i.e., a 27-week birth in the intervention arm and a 30-week birth in the placebo arm) was excluded, the excess neonatal deaths attributable to preterm birth was 21 (95%Crl: −20, 78) per 100,000 live births born to vaccinated mothers, and 0.69 (95%CrI: −0.66, 2.5) excess neonatal deaths may be associated with vaccination per every one infant life saved through protection against RSV through vaccination (Table 1). Lives saved exceed associated deaths in 66% of the simulations.

Excluding the five earliest births in each arm, the excess neonatal deaths would be 15 (95%CrI: −0.42, 39) per 100,000 live births born to vaccinated mothers and 0.48 (95%CrI: −0.014, 1.3) excess neonatal deaths may be associated with vaccination per every one infant life saved through vaccination. Lives saved exceed associated deaths in 92% of our simulations.

#### Vaccine efficacy.

If vaccine efficacy is assumed to remain constant at 80% throughout the first year of life, the estimated benefit in South Africa increases, but the point estimate would not substantially outweigh that of the risk (Section A2 in S2 Text). Further details of the sensitivity analyses are described in the Supplement.

## Discussion

If vaccination is introduced at 24–36 GA weeks, the estimated benefit of maternal vaccination through reduction in RSV-associated infant mortality is unlikely to substantially outweigh the potential risk of increased neonatal mortality due to vaccine-associated preterm birth in South Africa. However, there is considerable uncertainty around our modeled estimate of vaccine-related neonatal mortality risk. We also estimate that if vaccination is introduced at 27–36 GA weeks, the mortality benefit is likely to outweigh the risk. These findings are based on the speculation that the observed increased preterm birth was related to RSVpreF as being connected with the increased risk of preterm birth, based on observations in the South African component of the trial, and that this increased preterm birth risk leads to neonatal deaths based on GA-specific risks derived from the DCHS. The findings have been presented at the SAGE (the Strategic Advisory Group of Experts on Immunization) meeting in September 2024, and SAGE ultimately recommended RSV vaccination in the third trimester of pregnancy, as defined by the local context which in most countries is 28 GA weeks onwards [24]. Again, the observed association may not be causal, i.e., vaccination may not have caused the preterm births.

Our conclusions are largely dependent on a relatively small number of early preterm infants in the trial, and we find that the point estimate of the benefit-risk ratio could reverse if we exclude these early preterm infants in the trial. It is hard to compare these benefit-risk ratio with other vaccines’ ratios, which are context-specific [25–27]. In the scenario analysis, we ran one scenario in which the risk and benefit of vaccination were weighted equally. Meanwhile, we also ran another scenario in which the risk is weighted five times the benefit, given that debates regarding decision-making in vaccination; i.e., people may put more emphasis on the risk of vaccination than on the benefit [25,28,29]. The results are also subject to uncertainty surrounding limited data on mortality of early preterm infants. Furthermore, by applying the model-estimated neonatal mortality risks to the number of live births in the South African component of the trial, the findings indicate that expected excess deaths either in the benefit or the risk were too small to be detected among the small number of live births in the South African component of the trial, in which there was no increase in deaths based on vaccination. This analysis is based on limited data from a single country in the MATISSE trial. Given the limited number of participants in the single country, the observed numerical imbalance may simply be a Type I error, or could reflect a (currently unknown) biological mechanism. While our study cannot determine which is more likely, it extrapolates South African trial birth outcomes assuming the effect is genuine.

Maternal RSV vaccination with restricted GA windows has been licensed by several regulatory authorities in high-income countries (HICs) [18,30]. Notably, licensure indications for RSV immunization vary among HICs; e.g., EMA allows for maternal vaccination from 24 GA weeks onwards. In South Africa, the maternal vaccine has been licensed for use in pregnant women between 28 and 36 GA weeks, and the NITAG has recommended the vaccination during this period, i.e., from the third trimester onwards. Although there remain challenges to practicing this in LMICs (e.g., there is considerable uncertainty around GA assessment [31], timing of attendance to antenatal care [32], etc.), our analyses indicate that with vaccination from 27 weeks onward, the benefits may outweigh the risks. Our analyses and the subsequent SAGE recommendation support decision-makers in LMICs in introducing maternal RSV vaccination in their countries. Also, post-licensure surveillance is needed to monitor the association carefully in order to address concerns about potential causality between preterm birth and vaccination. There is ongoing post-marketing surveillance to assess potential adverse outcomes including preterm birth among vaccinees in early-introducing countries (e.g., U.S., Argentina, U.K.) [33,34]. In the U.S., among 13 healthcare organizations, a target trial emulation with matched analysis of data from the first year of vaccine use found the preterm birth rate was 4.0% among vaccinated and 4.5% among unvaccinated pregnant individuals (RR: 0.90; 95%CI: 0.80–1.00) [35]. Moreover, a multisite phase IV study is planned in Africa that will evaluate preterm births (NCT06955728) [36]. GA-specific neonatal mortality estimates will need to be updated once we have results from the phase IV study.

Our study design had several limitations. Firstly, our analysis focuses solely on mortality due to the impact of RSV and preterm birth. However, the burden of both conditions can extend beyond mortality. For instance, our previous analysis estimated that maternal vaccination would reduce RSV-associated hospitalizations in South Africa by 24.2% (95%CrI: 18.7, 28.6) and RSV-associated deaths by 27.4% (95%CrI: 21.6, 32.3) [8]. Both RSV-associated LRTI during early childhood and preterm birth have also been linked to long-term consequences. Although most of the total disease burden, as measured through disability-adjusted life years, is due to deaths, evaluating non-fatal and long-term outcomes would provide further refinement to the estimated benefit-risk ratio from vaccination. We also used estimates of reductions in RSV-associated infant deaths based on RSV disease burden in 2011–2016 in South Africa [4]. Also, we did not include analysis of the potential secondary benefits of prevention of severe RSV disease in infants through freeing up resources for other conditions (e.g., more availability of hospital beds, etc), enabling reduction of mortality from other treatable causes [37]. In addition, we estimated benefits using efficacy estimated from all births in the South African component of the trial (i.e., births born to mothers given intervention or placebo at 24–36 GA weeks). Meanwhile, for the 27–36 GA weeks analysis, we estimated risks using data from the subset of trial births: i.e., we used 27–36 GA weeks vaccinated (or given placebo) dataset for the 27–36 weeks analysis, while using 24–36 weeks dataset for the 24–36 weeks analysis because of limited data availability. This assumes that vaccine efficacy is consistent between mothers vaccinated at 24–36 weeks and those vaccinated at 27–36 weeks. Furthermore, in estimating the waning vaccine efficacy, we only considered the influence of time since birth. Hence potential influence of GA on vaccine efficacy is not captured in our analysis. Given that protection may be lower if infants are born within 14 days after vaccination [19,20] this study may overestimate or underestimate the benefits depending on the distribution of GA at vaccination in the population. We also assumed that uniform protection regardless of GA at birth when estimating the RSV-associated deaths averted through vaccination (benefit). Hence potential variation of protection between infants born term and preterm [19,20] is not captured in our analysis. This study may overestimate or underestimate the benefits depending on the distribution of GA at birth.

Another limitation is that we used the baseline preterm birth risk from the placebo arm of the trial, which is substantially lower than the overall preterm birth risk in South Africa [38,39]. For instance, Ohuma and colleagues estimated that preterm birth rate in 2020 is 13 (95%CI: 9.2, 17.9) per 100 live births in South Africa [39].

In addition, another limitation is that our conclusions were very sensitive to the outcomes associated with early preterm infants, but as the South African cohort data aggregated the neonatal mortality risk before 28 weeks, we did not know the exact GA-specific neonatal mortality risk before 28 weeks and instead assumed a constant risk. Moreover, our estimates of GA-specific neonatal mortality are based on a study conducted between 2012 and 2015 of a population-based birth cohort of unselected pregnant women attending public health facilities in a low income area of South Africa [16]. Using more recent neonatal survival rates may change our conclusions, depending on whether RSV management or general neonatal care in the study setting has improved more rapidly. Lastly, given the advanced capacity for neonatal and pediatric intensive care in South Africa, case-fatality from severe RSV disease, as well as early preterm births, is likely lower than in some other LMICs; thus, the findings of this model based on South African trial data might not be generalizable to some other LMIC settings.

It is currently not established nor understood whether the observed association between vaccination and preterm birth is genuine or causal [10,40].The numerical imbalance in preterm birth in the trial was only statistically significant in South Africa and occurred predominantly at peaks of the delta and omicron waves of SARS-CoV-2 [11]. A similar preterm birth imbalance was observed in a trial of another pre-F maternal vaccine also undertaken during the Covid-19 pandemic [12]; however, no imbalance in preterm births was observed in a pre-pandemic trial of another maternal RSV vaccine that enrolled over half of the participants in South Africa [41]. Moreover, in the MATISSE trial most infants were born more than 30 days after vaccination, and there was no temporal relationship or proposed biological mechanism between the vaccination and preterm birth [18].

Post-licensure surveillance is needed to clarify if RSVpreF and preterm birth are associated [11,14,42], however, it was out of the scope of our analysis. Establishment of safety monitoring was recommended by the World Health Organization (WHO) in countries where maternal RSV vaccine is to be introduced [20]. However, WHO position paper noted that the vaccine introduction should not wait until surveillance systems have been set up, while emphasizing the need for adequate funding, training and planning to support such activities. Our analysis did not consider some other key outcomes that are potentially important, including severe RSV disease associated with preterm birth [43], stillbirths, and other fetal deaths [10], or seasonal and other temporal variations in RSV incidence [4] and preterm birth risk [18].

Our study illustrates the potential importance of the observed imbalance in preterm birth following maternal RSV vaccination at broader GA vaccination windows. However, we also show that any potential risk could be largely mitigated by changing vaccine eligibility to begin in the third trimester. The first long-awaited maternal RSV vaccine has recently been recommended by WHO SAGE for use in the third trimester of pregnancy and will likely be globally available in the next few years. Post-marketing surveillance is important to obtain further evidence about its safety and effectiveness when used in real-world settings.

## Supporting information

S1 Text Supplementary text, including: Figs A–C and Tables A–I. (DOCX)

S2 Text Supplementary text, including: Figs A and B and Table A. (DOCX)

## Abbreviations

CFR: case fatality rate
CI: confidence interval
Crl: credible interval
DCHS: Drakenstein Child Health Study
EMA: European Medicines Agency
HICs: high-income countries
LMICs: low- and middle-income countries
LRTIs: lower respiratory tract infections
MA: medically-attended
MCMC: Markov chain Monte Carlo
SAGE: Strategic Advisory Group of Experts on Immunization
