# Supplementary material for: The benefits and risks of maternal RSV vaccination on mortality in South Africa: A modeling study
Source: PLoS Med. 2026 Jan 20;23(1):e1004625. doi: 10.1371/journal.pmed.1004625 (PMC12818683; doi:10.1371/journal.pmed.1004625)
Supplement: S1 Text — (DOCX) [file pmed.1004625.s001.docx]

# S1 Text

## A. Overview of modelling framework

Using static cohort models, we estimated under-1-year-old RSV-associated deaths averted through vaccination (benefit) and neonatal deaths potentially associated with preterm birth (risk). In order to compare the benefit and the risk, we calculated net mortality (= excess neonatal deaths (risk) – infant saved by vaccination (benefit)) per 100,000 live births born to vaccinated mothers, and the risk-benefit ratio (excess neonatal deaths (risk) per 1 infant saved by vaccination (benefit)).

## B. Estimates of benefit

### B1. Model outline

We estimated under-1-year-old RSV-associated deaths averted through vaccination (benefit) by stratifying all outcomes into age groups (from 0 to 11 months of age). We multiplied estimated RSV-associated deaths by estimated vaccine efficacy. Then we summed up the averted deaths over all age groups to calculate the benefit.

$$B= \sum_{i=0}^{11} (H_{i}* C_{i}* R_{i}* V_{i})$$

Here, *B* is under-1-year-old RSV-associated deaths averted by vaccination. *Hi* is estimated RSV-associated SARI hospitalization at age in months *i*, *Ci* is in-hospital CFR at age in months *i* and *Ri* is the ratio of out-of-hospital deaths and in-hospital deaths at age in months *i* obtained from [1,2]. *Vi* is the estimated vaccine efficacy at age in month *i*.

## C. Estimates of RSV-associated deaths

### C1. Key parameters

1. RSV-associated SARI hospitalization rate per 10,000 person-years by age group

| Age groups (month) | Median value (95% CI) |
| --- | --- |
| 0 | 6803.7 (5065.3, 8529.0) |
| 1 | 6831.7 (5858.1, 7841.9) |
| 2 | 4857.4 (4003.7, 5748.9) |
| 3 | 4307.5 (3459.4, 5191.3) |
| 4 | 4089.1 (3161.5, 5095.0) |
| 5 | 2603.8 (1921.5, 3405.2) |
| 6 | 2839.9 (2115.5, 3607.7) |
| 7 | 1919.7 (1282.1, 2605.0) |
| 8 | 1292.0 (804.9, 1766.1) |
| 9 | 1090.6 (622.7, 1661.7) |
| 10 | 1073.5 (629.4, 1581.2) |
| 11 | 1000.3 (577.5, 1494.5) |

1. In-hospital CFR by age group

| Age groups (month) | Value (%) |
| --- | --- |
| 0-5 | 1.21 |
| 6-11 | 1.08 |

1. Ratio of out-of-hospital deaths to in-hospital deaths by age group

| Age groups (month) | Value |
| --- | --- |
| 0-11 | 0.35 |

**Table A. Parameters for estimates of RSV-associated deaths. (A) RSV-associated SARI hospitalization rate per 10,000 person-years (B) in-hospital CFR by age group (C) Ratio of out-of-hospital to in-hospital deaths by age group.**

## D. Estimates of waning efficacy

### D1. Model outline

Observed disease outcomes in trial suggested that vaccine efficacy wanes after birth [3,4]. We modelled *VE_est(t)* vaccine efficacy at time t after birth as the product of *V0*, the initial value of efficacy, and *w(t),* the function of protection decay of efficacy after birth. We assumed that *w(t)* follows an Erlang-k distribution in which both the shape and scale parameters can be estimated [5].

$w\left( t \right)= \sum_{n=0}^{k-1} \frac{{T\_v}^{n}t^{n}e^{-T\_v * t}}{n!}$...(1)

Equation (1) shows the counter cumulative density function of Erlang-k distribution. We explored two typical protection decay functions (i.e., exponential distribution (k = 1), Erlang-2 distribution (k = 2)). We estimated the efficacy waning rate by fitting the modelled outcomes to the observed outcomes in the trial. The incidence in the intervention arm at time t after birth *I_v(t)* was estimated using modelled efficacy and the incidence in the placebo arm *I_p(t).*

*I_v (t) = I_p(t)**(1 - *VEest(t)*)

Since the trial outcomes were grouped into 30-day intervals from birth to 180 days after birth, the means of the estimated vaccine efficacy during 30-day period were used to calculate the number of infected individuals in intervention arm *I_v (t)* during that period. The modelled outcomes *C* (i.e., individuals newly counted as that disease outcome during that period) in the intervention arm were fitted to the RSV disease outcomes in the trial.

*C* *~ B*(*N*, *I_v (t_mid)*)

In the intervention arm*, N* is the total number of individuals in the arm, and *I_v (t_mid)* is the incidence in the intervention arm in the middle of that period assuming incidence is constant during that period. We assume that vaccine efficacy against RSV disease outcomes with different severities wanes at the same rate but the initial values *V0* are severity-dependent. Data analysis code is published on https://github.com/ayakamon/BR-RSV-MV.

### D2. Overview of data

We used the final outcome of the trial (Table S2), in which the vaccine showed efficacy against RSV disease in infants born to vaccinated mothers [4]. We calculated incremental efficacy from data on cumulative counts of severe RSV-Positive MA-LRTIs (Table B(A) in S1 Text) and less severe RSV-Positive MA-LRTIs (Table B(B) in S1 Text) from 0 to 180 days after birth in each trial arm across the entire study.

1. Cumulative counts of severe RSV-positive MA-LRTIs through 180 days of age

| Time after birth (days) | RSVpreF 120μg  N = 3585 | Placebo  N = 3563 |
| --- | --- | --- |
| 0 - 30 | 1 | 10 |
| 30 – 60 | 4 | 28 |
| 60 – 90 | 6 | 34 |
| 90 - 120 | 13 | 49 |
| 120 - 150 | 18 | 61 |
| 150 - 180 | 21 | 70 |

1. Cumulative counts of less severe RSV-positive MA-LRTIs through 180 days of age

| Time after birth (days) | RSVpreF 120μg  N = 3585 | Placebo  N = 3563 |
| --- | --- | --- |
| 0 - 30 | 2 | 15 |
| 30 – 60 | 14 | 38 |
| 60 – 90 | 25 | 59 |
| 90 - 120 | 40 | 88 |
| 120 - 150 | 55 | 110 |
| 150 - 180 | 67 | 132 |

**Table B. Cumulative number of trial outcomes by 30-day interval from 0 to 180 days after birth regarding (A) severe RSV-Positive MA-LRTIs and (B) less severe RSV-Positive MA-LRTIs** [4]**.**

### D3. Model fitting

The numbers of counts of severe RSV-Positive MA-LRTIs and less severe RSV-Positive MA-LRTIs were fitted with a binomial likelihood. The Erlang-2 model was fitted using a Metropolis-Hastings Sampler implemented in the R package BayesianTools [6] with an iteration of 10,000 samples and thinned by 10, then 1,000 samples were used for the benefit calculation. Data analysis code published on <https://github.com/ayakamon/BR-RSV-MV>.

| Symbol | Parameter | Prior |
| --- | --- | --- |
| VE0_s | Vaccine efficacy against severe disease at birth | Uniform(0.001, 0.99) |
| VE0_l | Vaccine efficacy against less severe disease at birth | Uniform(0.001, 0.99) |
| T_v | Rate of Erlang-2 distribution | Uniform(0.001, 0.99) |

**Table C. Parameters of waning efficacy of RSVpreF.**

### D4 Fitted results

To model vaccine waning, we compared an exponential distribution model an Erlang-2 distribution and a Weibull distribution (Fig A in S1 Text). Among the best fit models (i.e., Erlang-2 distribution and exponential distribution) (Table D in S1 Text), we chose Erlang-2 distribution as the more conservative estimate in an attempt to avoid overestimating protection beyond the observed data. Effective sample size (ESS) for each parameter in Erlang-2 distribution model is shown in Table E in S1 Text. To estimate RSV-associated infant deaths averted by vaccination (benefit), we used modelled efficacy against severe outcomes during the first year of life.


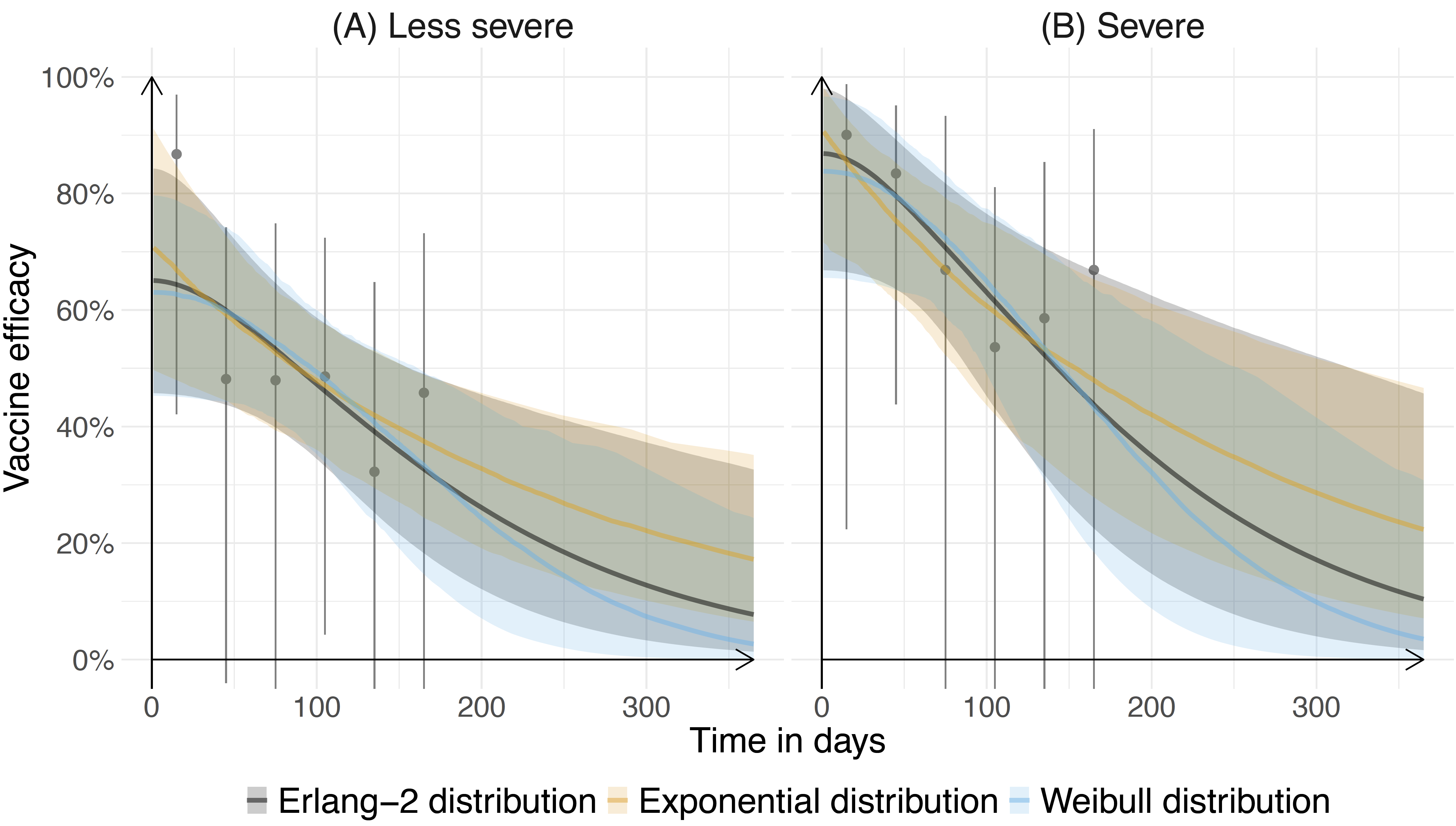


**Fig A.** **Vaccine efficacy of RSVpreF against RSV-associated (A) less severe and (B) severe MA-LRTIs during the first year of life.** Vaccine efficacy against RSV-associated severe MA-LRTIs observed in the trial is shown as gray dots together with binomial 95% confidence intervals. Modelled efficacy is shown as a gray line (Erlang-2 distribution model), an orange line (Exponential distribution model) and a blue line (Weibull distribution model) with shaded area of 95% credible intervals.

| Distribution | WAIC |
| --- | --- |
| Erlang-2 | 53.5 |
| Exponential | 52.0 |
| Weibull | 55.5 |

**Table D. Watanabe-Akaike Information Criterion (WAIC) according to different distributions for estimating waning efficacy of RSVpreF.**

| Symbol | Parameter | ESS |
| --- | --- | --- |
| VE0_s | Vaccine efficacy against severe disease at birth | 665.7147 |
| VE0_l | Vaccine efficacy against less severe disease at birth | 788.0205 |
| T_v | Rate of Erlang-2 distribution | 639.0123 |

**Table E. Effective sample size (ESS) for** **parameters of waning efficacy of RSVpreF.**


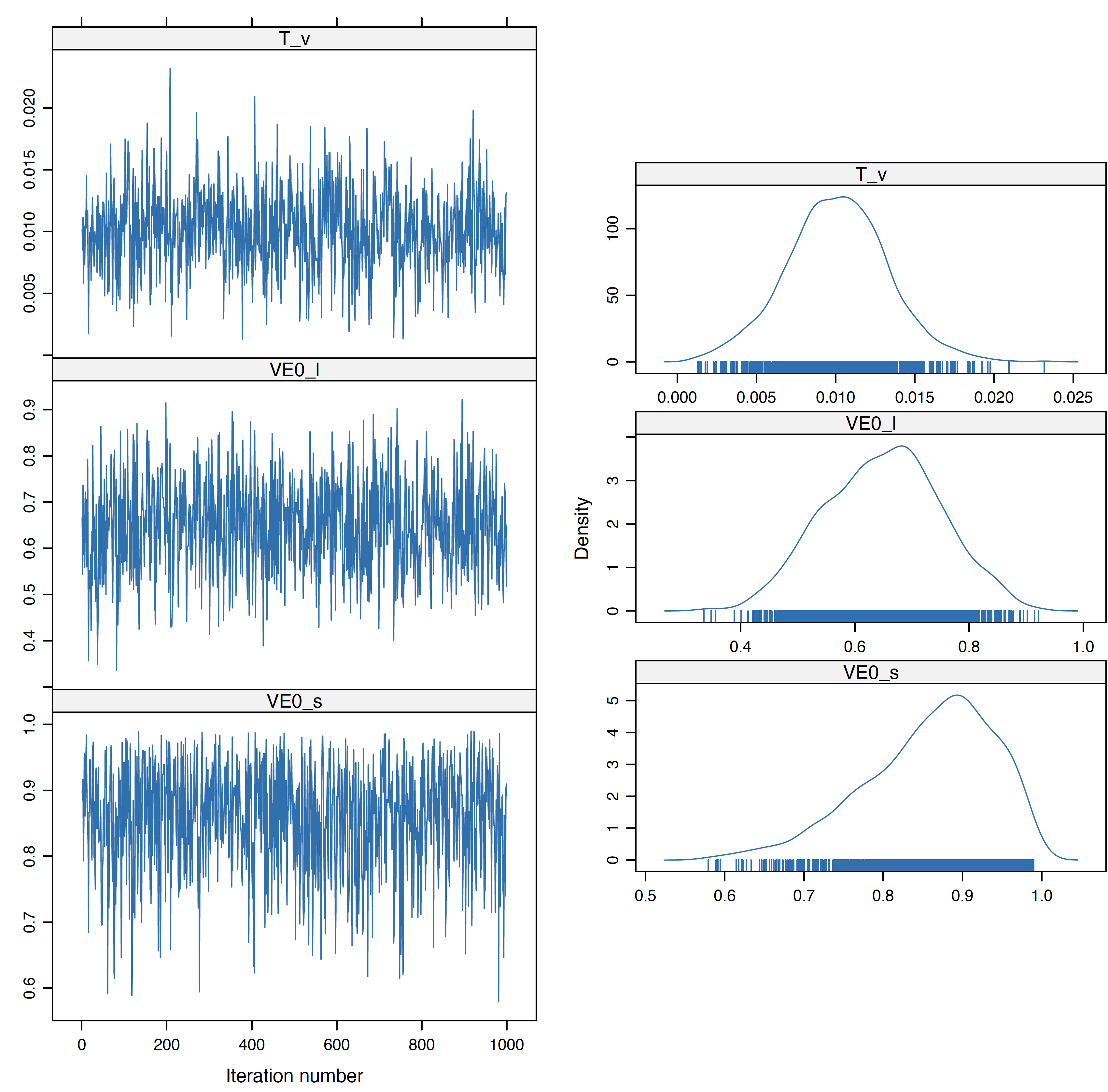


**Fig B. Trace plots (left) and density plot (right) of fitted model by parameter.** Each parameter corresponds to the parameters in Table E.

## E. Estimates of risk

### E1. Model outline

We estimated neonatal deaths due to preterm birth potentially associated with vaccination (risk) by stratifying outcomes into GA groups (<28 weeks, 28, 29, 30, 31, 32, 33, 34, 35, 36, and 37+ weeks gestation). We multiplied estimated GA-specific neonatal mortality by GA-specific difference in proportion of births born to vaccinated mothers and mothers given placebo. Then we summed up to calculate the risk.

$$R= \sum_{i} (N_{i}* P_{i})$$

Here, *R* is neonatal deaths due to preterm births potentially associated with vaccination. *Ni* is estimated neonatal mortality of infants born at GA *i* weeks, *Pi* is the difference between proportional risk of births born at GA *i* weeks to vaccinated mothers and mothers given placebo.

## F. Estimates of GA-specific neonatal mortality

### F1. Model outline

Observed neonatal mortality in the South African cohort study is proportionately high among infants born at earlier gestational age compared to infants born at later gestational age. We assumed that GA-specific neonatal mortality depends on GA.

*D ~ B(B,* $N_{i}$*)*

*D* is the modelled outcomes (i.e., number of neonatal deaths among infants born in that GA group). *B* is the number of births in that GA group, and *Ni* is the modelled neonatal mortality in that GA group.

The neonatal mortality of infants born before 28 GA weeks is assumed to be constant and the same as that at 27 GA weeks, because of lack of disaggregated data.

For infants born at 28-36 GA weeks, we modelled neonatal mortality of infants born in each GA group *N(t)* assuming it follows an Erlang-2 distribution.

　$N\left( t \right)={NMR}_{0}* \sum_{n=0}^{1} \frac{{T\_m}^{n}t^{n}e^{-T\_m*t}}{n!}$...(2)

t in equation (2) is time after 27 GA weeks. *NMR0* is neonatal mortality of infants born in GA group <28 weeks. *T_m* is the rate of Erlang-2 distribution.

For infants born after 36 weeks, we also assumed that neonatal mortality risk was constant from 37 weeks onwards.

### F2. Model fitting

GA-specific neonatal mortality in South Africa was inferred by fitting the model to GA-specific number of births and neonatal deaths in the South African cohort study [7] in a Bayesian framework. The numbers of deaths were fitted with a binomial likelihood. The model was fitted using a Metropolis-Hastings Sampler implemented in the R package BayesianTools [6] with an iteration of 50,000 samples and a burn-in of 1,000 samples thinned by 4, then 10,000 samples are used for the risk calculation. Effective sample size (ESS) for each parameter is shown in Table G in S1 Text.

Data analysis code is published on <https://github.com/ayakamon/BR-RSV-MV>.

| Symbol | Parameter | Prior |
| --- | --- | --- |
| NMR0 | Neonatal mortality of infants born in the GA group <28 weeks | Uniform(0.1, 0.5) |
| T_m | Rate of Erlang-2 distribution | Uniform(0.01, 0.1) |

**Table F**. **Parameters of GA-specific neonatal mortality in the South African cohort study.**

### F3. Fitted results

| Symbol | Parameter | ESS |
| --- | --- | --- |
| NMR0 | Neonatal mortality of infants born in the GA group <28 weeks | 1262.807 |
| T_m | Rate of Erlang-2 distribution | 1548.331 |

**Table G**. **Effective sample size (ESS) for** **parameters of GA-specific neonatal mortality in the South African cohort study.**


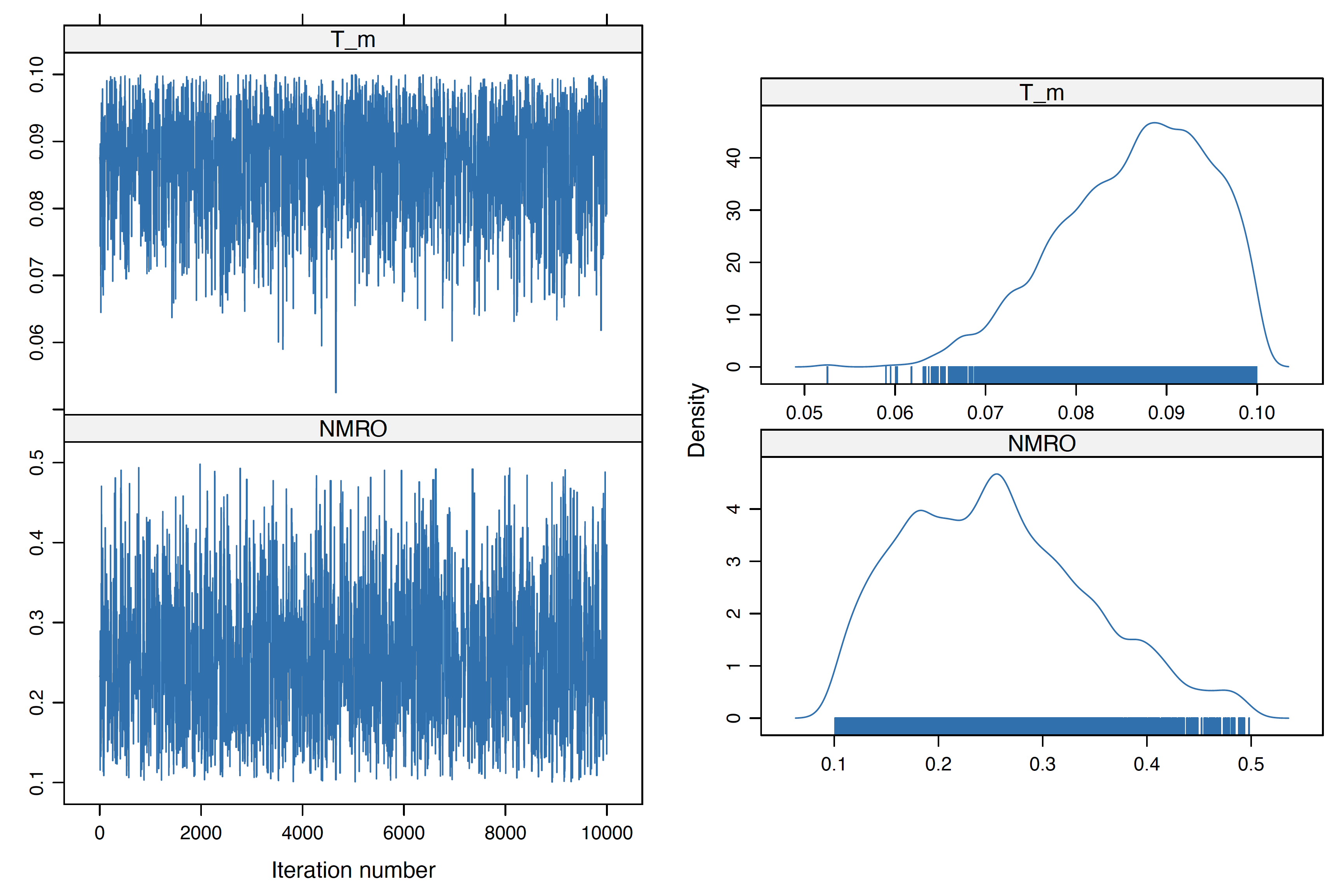


**Fig C. Trace plots (left) and density plot (right) of fitted model by parameter.** Each parameter corresponds to the parameters in Table G.

## G. GA-specific risk of births born to mothers by trial arm

| GA | Trial arm | Number of births |
| --- | --- | --- |
| 27 | vaccine | 1.4 |
| 30 | placebo | 0.9 |
| 31 | vaccine | 1.4 |
| 31 | placebo | 0.9 |
| 32 | vaccine | 1.4 |
| 33 | vaccine | 2.3 |
| 33 | placebo | 0.9 |
| 34 | vaccine | 5.3 |
| 34 | placebo | 5.5 |
| 35 | vaccine | 10.1 |
| 35 | placebo | 1.8 |
| 36 | vaccine | 19.3 |
| 36 | placebo | 7.8 |
| 37 | vaccine | 29.1 |
| 37 | placebo | 30.7 |
| 38 | vaccine | 83.3 |
| 38 | placebo | 88.8 |
| 39 | vaccine | 119.1 |
| 39 | placebo | 122.7 |
| 40 | vaccine | 135.1 |
| 40 | placebo | 128.7 |
| 41 | vaccine | 53.4 |
| 41 | placebo | 66.5 |
| 42 | vaccine | 9.4 |
| 42 | placebo | 10.6 |
| 43 | vaccine | 1.4 |
| 43 | placebo | 0.9 |

**Table H**. **GA-specific births born to mothers vaccinated or given placebo at 24-36 GA weeks in the South African component of the trial by trial arm.** The table shows data obtained by digitizing figures in [8].

| GA | Trial arm | Number of births |
| --- | --- | --- |
| 30 | placebo | 0.9 |
| 31 | placebo | 0.9 |
| 33 | vaccine | 2.4 |
| 33 | placebo | 0.9 |
| 34 | vaccine | 4.1 |
| 34 | placebo | 4.8 |
| 35 | vaccine | 6.1 |
| 35 | placebo | 0.9 |
| 36 | vaccine | 17.4 |
| 36 | placebo | 2.6 |
| 37 | vaccine | 23.1 |
| 37 | placebo | 19.2 |
| 38 | vaccine | 65.0 |
| 38 | placebo | 64.1 |
| 39 | vaccine | 92.4 |
| 39 | placebo | 85.9 |
| 40 | vaccine | 100.1 |
| 40 | placebo | 99.0 |
| 41 | vaccine | 39.2 |
| 41 | placebo | 50.1 |
| 42 | vaccine | 9.2 |
| 42 | placebo | 7.8 |
| 43 | placebo | 0.9 |

**Table I**. **GA-specific births born to mothers vaccinated or given placebo at 24-36 GA weeks in the South African component of the trial by trial arm.** The table shows data obtained by digitizing figures in [8].

# References:

1. Koltai M, Moyes J, Nyawanda B, Nyiro J, Munywoki PK, Tempia S, et al. Estimating the cost-effectiveness of maternal vaccination and monoclonal antibodies for respiratory syncytial virus in Kenya and South Africa. BMC Med. 2023 Mar 31;21(1):120.

2. Cohen C, Walaza S, Treurnicht FK, McMorrow M, Madhi SA, McAnerney JM, et al. In- and Out-of-hospital Mortality Associated with Seasonal and Pandemic Influenza and Respiratory Syncytial Virus in South Africa, 2009–2013. Clinical Infectious Diseases. 2018 Jan 6;66(1):95–103.

3. Kampmann B, Madhi SA, Munjal I, Simões EAF, Pahud BA, Llapur C, et al. Bivalent Prefusion F Vaccine in Pregnancy to Prevent RSV Illness in Infants. N Engl J Med. 2023 Apr 20;388(16):1451–64.

4. Munjai I. Protection Against Infant Illness with a Bivalent RSVpreF Vaccine in Pregnancy: Final Analysis. RSVVW’24; 2024 Feb 15; Mumbai.

5. Hodgson D, Wilkins N, Van Leeuwen E, Watson CH, Crofts J, Flasche S, et al. Protecting infants against RSV disease: an impact and cost-effectiveness comparison of long-acting monoclonal antibodies and maternal vaccination. The Lancet Regional Health - Europe. 2024 Jan;100829.

6. Hartig F, Minunno F, Paul S, Cameron D, Ott T, Pichler M. BayesianTools: General-Purpose MCMC and SMC Samplers and Tools for Bayesian Statistics. 2023 Jan 30; Available from: https://cran.r-project.org/web/packages/BayesianTools/index.html

7. Zar HJ, Pellowski JA, Cohen S, Barnett W, Vanker A, Koen N, et al. Maternal health and birth outcomes in a South African birth cohort study. Hill B, editor. PLoS ONE. 2019 Nov 21;14(11):e0222399.

8. Monoi A, Endo A, Procter S, Leuba S, Flasche S, Jit M, et al. Benefit-risk analysis of maternal vaccination in South Africa [Internet]. Hybrid presented at: SAGE meeting. Strategic Advisory Group of Experts on Immunization 23-26 September 2024; 2024 09 [cited 2024 Oct 9]; Geneva, Switzerland. Available from: https://terrance.who.int/mediacentre/data/sage/SAGE_Slidedeck_September-2024.pdf.
