# Supplementary material for: The benefits and risks of maternal RSV vaccination on mortality in South Africa: A modeling study
Source: PLoS Med. 2026 Jan 20;23(1):e1004625. doi: 10.1371/journal.pmed.1004625 (PMC12818683; doi:10.1371/journal.pmed.1004625)
Supplement: S2 Text — (DOCX) [file pmed.1004625.s002.docx]

# S2 Text

## A. Estimated results

### A1. Scenario analyses on trial births


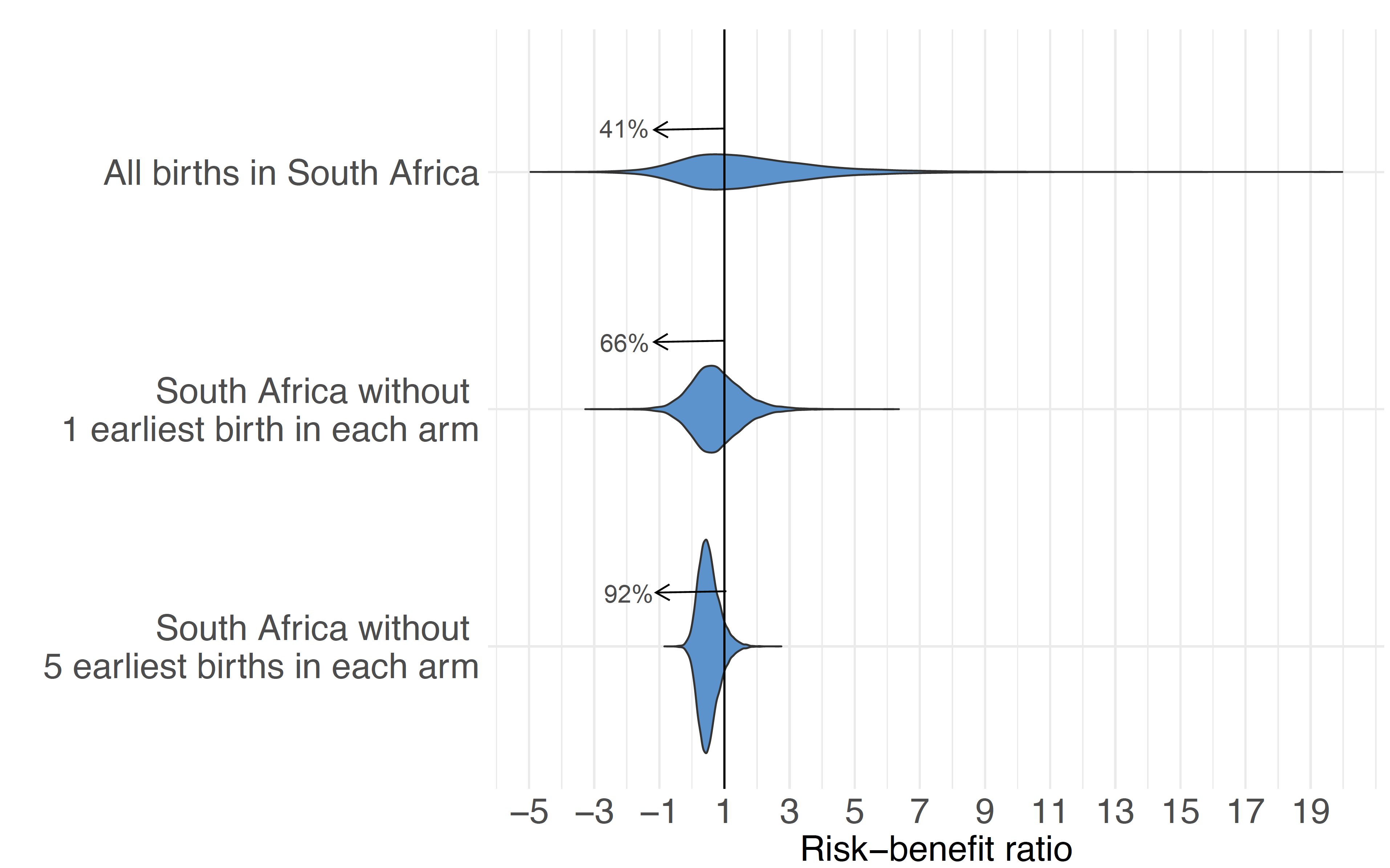


**Fig A.** **Estimated risk-benefit ratio of RSVpreF in South Africa under different scenarios on trial birth outcomes.** The plots illustrate excess neonatal deaths potentially associated with vaccination per every one infant under 1 year saved through vaccination (i.e., the ratio between the risk and benefit). The risk is measured by the excess neonatal deaths potentially attributable to vaccine-associated preterm birth per 100,000 live births born to vaccinated mothers and uses bootstrapped outcomes with 10,000 iterations. The benefit is measured by the vaccine-preventable RSV-associated deaths in less than 1 year-old infants per 100,000 live births born to vaccinated mothers and uses bootstrapped outcomes with 10,000 iterations. When the ratio is below 1 (black vertical line), the estimated benefits exceed the estimated risks. The risks are estimated using data in the South African component of the trial with all births, births in the South African component of the trial without the first earliest birth in each arm (i.e., 27 weeks in the intervention arm and 30 weeks in the placebo arm), or births in the South African component of the trial without the 5 earliest births in each arm. The percentages beside each plot show the percentage of simulations when the benefit exceeds the risk.


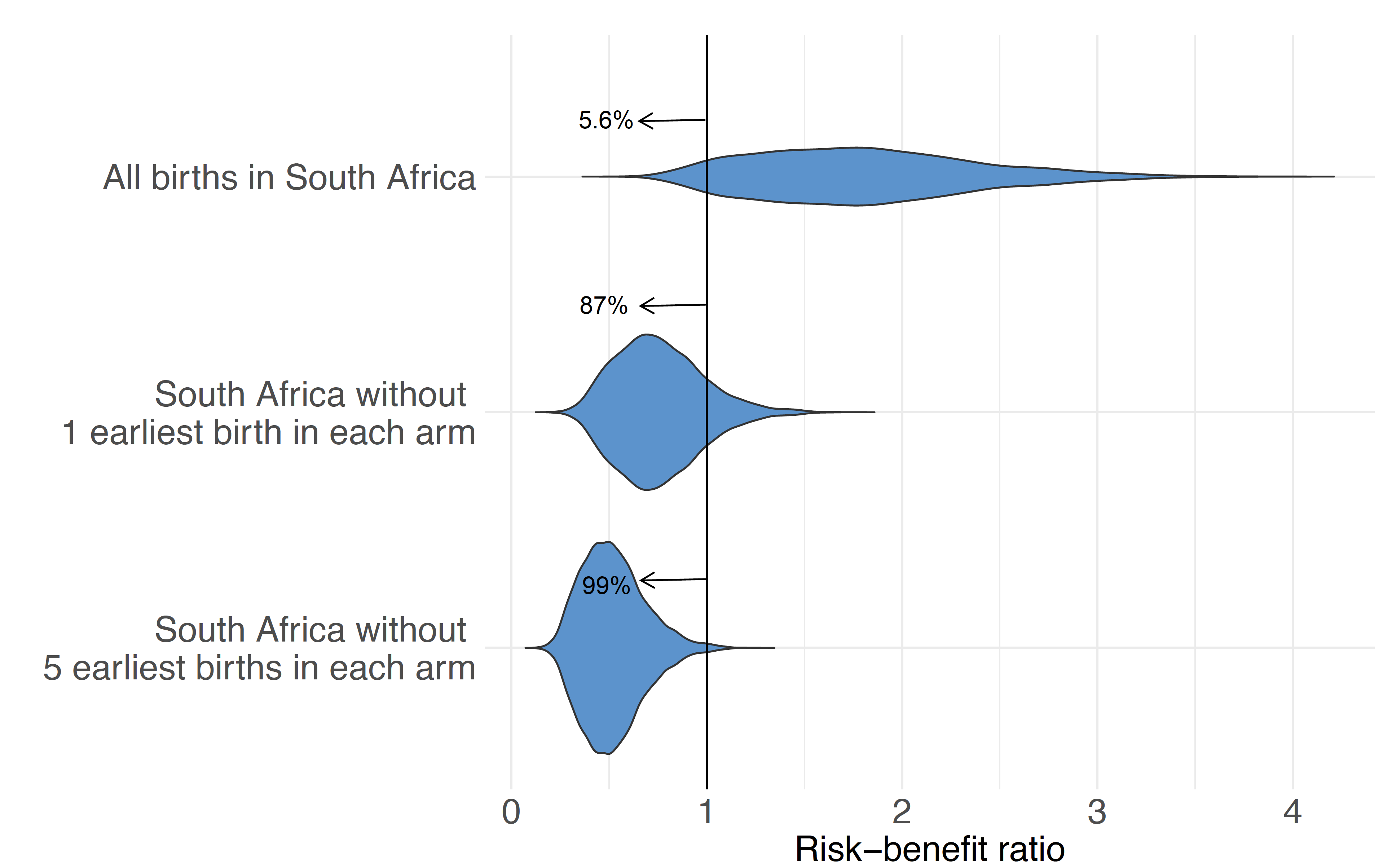


**Fig B. Estimated risk-benefit ratio of RSVpreF in South Africa under different scenarios on trial birth outcomes without incorporating uncertainty in trial outcomes.** The risk is measured by the excess neonatal deaths potentially attributable to vaccine-associated preterm birth and uses the trial birth outcomes without bootstrapping. The benefit is measured by the vaccine-preventable RSV-associated deaths in less than 1 year-old infants per 100,000 live births born to vaccinated mothers and uses bootstrapped outcomes with 10,000 iterations. When the ratio is below 1 (black vertical line), the estimated benefits exceed the estimated benefits. The risks are estimated using all birth outcomes from the South Africa component in the trial, births in the South Africa component without the first earliest birth in each arm (i.e., 27 weeks in the intervention arm and 30 weeks in the placebo arm), and births in the South African component of the trial without the 5 earliest births in each arm.

### A2. Vaccine Efficacy

With an optimistic assumption around vaccine efficacy that vaccine efficacy against severe RSV disease remains at 80% during the first year of life without any waning, the estimated benefit in South Africa increases from the waning vaccine assumption of 31 (95%CrI: 27, 35) to 41 (95%CrI: 38, 44) vaccine-preventable RSV-associated infant deaths per 100,000 live births born to vaccinated mothers.

### A3. Neonatal mortality of neonates born after 36 weeks

Instead of assuming constant neonatal mortality of neonates born after 36 GA weeks, we conducted a scenario analysis using birth data from VNMC, a study combining population-based data from 15 LMICs [1]. We used GA-specific neonatal mortality data from VNMC for live births born after 36 GA weeks divided by the overall neonatal mortality after 36 GA weeks from VNMC. Then we calculated the total number of deaths per total number of live births born after 36 GA weeks from DCHS. Then we multiplied the mean mortality of live births born after 36 GA weeks from DCHS by the standardized GA-specific neonatal mortality for each GA after 36 GA weeks from VNMC. We then multiplied the VNMC-adjusted DCHS GA-specific deaths divided by live births by the bootstrapped birth outcomes in the trial to estimate the excess neonatal deaths potentially associated with vaccination. We then estimated that the excess neonatal deaths potentially associated with vaccination would be 51 per 100,000 live births born to vaccinated mothers. Compared to the risk estimates assuming that all the deaths observed in the South African cohort study were only among infants born at 37 weeks and that neonatal mortality was constant after 36 weeks, the estimated neonatal mortality is generally smaller (e.g., for infants born at 37 GA weeks, 260 per 100,000 live births). Allowing neonatal mortality risk to vary after 36 weeks (as described above) results in a slight increase in excess deaths among vaccinees compared to controls (i.e., excess deaths increased from 44 per 100,000 live births born to vaccinated mothers from the main analysis, to 51 per 100,000 live births born to vaccinated mothers).

### A4. GA dating

GA dating by ultrasonography was performed at the second trimester in the South African cohort study [2]. However, this technique is believed to be only accurate within a margin of ±14 days [3]. We can incorporate this margin of error to further increase the uncertainty around GA at birth, but this has little effect on the point estimates of the risks of vaccination, or the conclusion that benefits are unlikely to outweigh the risks of neonatal deaths if vaccination is provided between 24-36 GA weeks.

| Excess deaths per 100,000 live births  born to vaccinated mothers (95%Crl) | Excess deaths per life saved through vaccination (95%Crl) |
| --- | --- |
| 52 (-45, 200) | 1.3 (-1.5, 6.7) |
| 54 (-47, 210) | 1.4 (-1.5, 7.0) |
| 49 (-44, 190) | 1.3 (-1.4, 6.3) |
| 48 (-41, 190) | 1.3 (-1.3, 6.1) |
| 48 (-39, 190) | 1.2 (-1.3, 6.2) |

**Table A**. **Estimated excess neonatal deaths using outcomes in Zar et al. with bootstrapping on GA dating.** 5 sets of results from the bootstrap are shown.

# References:

1. Hazel EA, Erchick DJ, Katz J, Lee ACC, Diaz M, Wu LSF, et al. Neonatal mortality risk of vulnerable newborns by fine stratum of gestational age and birthweight for 230 679 live births in nine low‐ and middle‐income countries, 2000–2017. BJOG. 2024 Jan 16;1471-0528.17743.

2. Zar HJ, Pellowski JA, Cohen S, Barnett W, Vanker A, Koen N, et al. Maternal health and birth outcomes in a South African birth cohort study. Hill B, editor. PLoS ONE. 2019 Nov 21;14(11):e0222399.

3. Methods for Estimating the Due Date. the American College of Obstetricians and Gynecologists’ Committee on Obstetric Practice; 2017 May. Report No.: Number 700.
